# Advances toward regenerative medicine in the central nervous system: challenges in making stem cell therapy a viable clinical strategy

**DOI:** 10.1186/2052-8426-2-12

**Published:** 2014-05-01

**Authors:** Elizabeth A Stoll

**Affiliations:** Institute of Neuroscience, Newcastle University, Framlington Place, Newcastle upon Tyne, UK

**Keywords:** Regenerative medicine, Stem cell therapy, Stem cell transplant, Progenitor cell, Neurodegenerative disease, Spinal cord injury

## Abstract

Over recent years, there has been a great deal of interest in the prospects of stem cell-based therapies for the treatment of nervous system disorders. The eagerness of scientists, clinicians, and spin-out companies to develop new therapies led to premature clinical trials in human patients, and now the initial excitement has largely turned to skepticism. Rather than embracing a defeatist attitude or pressing blindly ahead, I argue it is time to evaluate the challenges encountered by regenerative medicine in the central nervous system and the progress that is being made to solve these problems. In the twenty years since the adult brain was discovered to have an endogenous regenerative capacity, much basic research has been done to elucidate mechanisms controlling proliferation and cellular identity; how stem cells may be directed into neuronal lineages; genetic, pharmacological, and behavioral interventions that modulate neurogenic activity; and the exact nature of limitations to regeneration in the adult, aged, diseased and injured CNS. These findings should prove valuable in designing realistic clinical strategies to improve the prospects of stem cell-based therapies. In this review, I discuss how basic research continues to play a critical role in identifying both barriers and potential routes to regenerative therapy in the CNS.

## Review

### Introduction

Great strides in stem cell biology have been made in recent years, raising hopes for the development of individualized regenerative therapies to reverse cell loss and improve the function of tissues damaged by disease or trauma. Indeed a number of clinical trials are currently in progress to assess stem cell-based therapies for a range of conditions. Yet the central nervous system (CNS) poses unique problems to achieving safe, effective regenerative therapy. The vast majority of cells in the mammalian brain, eye, and spinal cord are produced during embryonic development, and these tissues are not disposed to regeneration in adult life. In short, the production of subtype-specific neurons, followed by the functional integration of these new cells into an existing three-dimensional structure with mature electrical and chemical properties, remains a great technical challenge.

The CNS was in fact long thought to be incapable of regeneration, but in the mid-1990s, cells were discovered in the adult brain with the capacity to self-renew and differentiate into various neuronal lineages [1, 2]. The discovery of more limited neural and glial progenitor cells residing in the brain, spinal cord, and eye soon followed [3, 4]. Before this discovery, in 1987, a small number of patients began receiving transplants of fetal brain tissue to replace cells lost in Parkinson’s Disease, with initially promising results [5]. A number of Phase I and II trials have been undertaken in the ensuing years to evaluate the safety and efficacy of stem cell transplants for the treatment of Alzheimer’s Disease, Huntington’s Disease, motor neuron disorders, stroke, macular degeneration, and spinal cord injury [6, 7]. Yet these trials have largely reported modest benefits, when results are reported at all. Disappointingly, the first controlled clinical trial using stem cells to treat spinal cord injury, initiated by Geron Corporation, was abruptly halted in November 2011, and other trials for stem cell therapies in the CNS have also been prematurely terminated. Phase I/II trials for implantation of purified human fetal neural stem cells in patients with spinal cord injury are however currently being conducted by Stem Cells, Inc. [7]. In addition, a number of trials are ongoing to implant stem cells - derived from fetal brain, umbilical cord blood, or adult bone marrow - into patients with ischemic stroke, amyotrophic lateral sclerosis, and other neurological conditions [8].

Although these therapies have not yet realized clinical success, hope should not be lost. A great deal has been discovered in the intervening period regarding the harnessing of regenerative potential. We now have a much greater understanding of mechanisms controlling proliferation and cellular identity; how stem cells may be directed into neuronal lineages; genetic, pharmacological, and behavioral interventions that modulate neurogenic activity; and the exact nature of limitations to regeneration in the adult, aged, diseased and injured CNS. This basic research has yielded both an understanding of the challenges encountered by stem cell therapies in the central nervous system and tools to tackle these concerns.

Below I discuss the restrictions to new neuron production in the adult mammalian CNS, largely gained from studying endogenous neural stem and progenitor cells (NSPCs) in the developing and adult brain. I then describe how neurogenic activity can be modulated *in vivo* to promote new neuron production. Next I highlight the problems posed to cell transplantation by the properties of the central nervous system and how these issues are being addressed within the field. Finally, I compare the prospects of cell replacement therapies with other methods to restore function to neural tissues affected by trauma or disease. In this review, I aim to articulate how basic research continues to play a critical role in identifying both barriers and potential routes to regenerative therapy in the CNS.

### Maintaining neurogenesis: lessons from the developing brain

Neurons are primarily produced in the mammalian brain during a short period of embryonic development (~E10-E14 in rodents), with the exception of cerebellum, where the majority of neurons are born in the early postnatal period. In fact, very few new cells are endogenously produced in the brain and spinal cord post-natally, and most new cells produced are glia (reviewed by [9]). There are two main areas of the adult mammalian CNS containing stem-like cells that retain neurogenic potential: the dentate gyrus of the hippocampus (DG), which produces new granule neurons that may play a role in learning and memory; and the subventricular zone (SVZ), which produces dopaminergic granule cells and GABAergic interneurons that migrate along the rostral migratory stream to populate the olfactory bulb (reviewed by [10]). Lower levels of neurogenesis, indicated by thymidine analog incorporation or progenitor cell-specific protein expression, are observed in the adult piriform cortex, striatum, hypothalamus, and amygdala [11–13]; some of these actively dividing neural progenitors originate from cells within the SVZ and travel along a temporal migratory stream [14].

During development, the neocortical layers are generated in a strictly controlled spatio-temporal pattern; the deepest layers are laid down first, and later-born neurons migrate past early-born neurons to generate the more superficial layers (reviewed in [15]). The process of fate restriction is controlled by sequential expression of transcription factors; this process is largely cell-intrinsic, as progenitors that normally produce upper-layer neurons (layers 2/3) cannot produce deep-layer Otx1+ neurons (layers 4–6) in a heterochronic transplant model [16]. Most cells in the cortex are thought to arise from the ventricular zone or SVZ, expressing layer-specific markers as they are generated, such as Fezl in corticospinal motor neurons and Cux2 in commissural and associative projection neurons [17–19]. Inhibitory cortical neuron lineages are independently generated, largely migrating from the medial ganglionic eminence [20, 21]. Meanwhile, medium spiny neurons within striatum arise from the lateral ganglionic eminences [21] and are characterized by their own unique patterns of gene expression [22]. The range of neuronal lineages produced by the adult brain is much more restricted than the range produced during development. For example, markers specific to neurons within superficial cortical layers, such as Svet1 and Cux2, can be found in the SVZ, suggesting that the adult brain can cultivate these types of cortical neurons but not neurons within deeper layers [18, 19]. This complex array of specific gene expression must be recapitulated in a therapeutic setting, either prior to or post-transplantation.

Other important differences between the highly proliferative developing brain and the less plastic adult brain lie in their respective biophysical properties. The developing SVZ is comprised of radial glia, cells which span the cortex and are used as scaffolding to assist migration of new neurons to appropriate locations within cortex [23]. The elastic properties of radial glia aid the new cells on their journey to their final location, and any disruption to the springiness of radial glial processes harms not only migratory activity but also neuronal maturation, as these actions are coordinated within the differentiating cell. Areas targeted for transplantation within the adult brain do not possess such unique biophysical properties and migratory paths which foster cellular integration.

Once the six layers of cortex are established, the developmentally-active ventricular zone is exhausted and the SVZ is separated from the lateral ventricles only by a thin layer of multi-ciliated, CD133+ ependymal cells. These largely quiescent, GFAP + Nestin + Dcx- stem-like cells (called B-type) intercalate into the ependymal layer as they divide. These divisions produce a highly proliferative GFAP-Nestin + Dcx + progenitor cell (called C-type); this progenitor in turn yields two or more lineage-restricted, transit-amplifying, neuronal precursors (called A-type), which migrate along the rostral migratory stream to the olfactory bulb (reviewed in [24]). During migration, the cells cease cell division and begin to mature into subtype-specific neurons. The process of cell cycle lengthening and exit is tightly coupled to lineage restriction and neuronal differentiation [25]. This whole process allows limited regeneration along a defined path in the adult mammalian brain.

A similar proliferative capacity exists in the adult hippocampal dentate gyrus. Development of the hippocampal formation begins at E15 in mice [26], and involves a succession of cellular matrices. The tertiary matrix is established by P5, and remains in place for the remainder of the lifespan. In the adult dentate gyrus, cells proliferate in the inner granule cell layer, and migrate radially outward as they differentiate [27]. New granule cells in hippocampal dentate gyrus receive primary glutamatergic afferents from entorhinal cortex and project axons to inhibitory interneurons and pyramidal cells of area CA3 (including the place cells originally characterized by O’Keefe and Dostrovsky). Immature neurons are highly excitable, so newly-born cells are thought to affect the dynamic properties of existing networks to which they are recruited [28]. Such circuit complexity must be kept in mind when considering cell transplantation in the CNS.

### The limitations of neurogenesis in the adult brain

Although new neurons are produced in these two discrete locations in the adult mammalian brain, neurogenesis in both areas significantly declines across the lifespan [2]. Age-associated decreases in neurogenesis are largely due to a depletion of the population of self-renewing cells, which is drastically reduced at the completion of brain development and further reduced across the lifespan [29, 30]. The remaining pool of neural stem and progenitor cells in the aged brain are characterized by a high mutational load [31] and dysregulated cell cycle kinetics [32]. Survival of newly-born neurons is low in adulthood and decreases further with age, likely due to both intrinsic factors and an inhospitable micro-environment [30].

Age-related decline in regenerative potential is a potential problem for stem cell-based therapies. With age, the CNS environment becomes less cordial to neurons and more conducive to glia; this is partially due to the propensity of aging NSPCs to preferentially differentiate into glial lineages over neuronal lineages [30]. However, the aging environment also appears to play a role: the brains of aged mice are characterized by striking increases in astrocytes and microglia [32]. In addition, chemokines present in the aged brain negatively regulate neurogenesis [33]. Yet even in the young adult brain, environmental cues appear to hinder neurogenesis except in the privileged areas of SVZ and DG. Several studies have attempted to transplant embryonic and postnatal NSPCs into non-neurogenic areas of the brain, with varying success in integration and adoption of locale-specific traits [34–37]. Meanwhile, glial progenitor cells from the spinal cord transplanted into hippocampal DG readily acquire a granule neuron phenotype, demonstrating that neurogenic zones have the capacity to reprogram otherwise lineage-restricted cells [38]. Unfortunately non-neurogenic areas of the adult CNS, where cell transplants are most desirable, usually prohibit the incorporation of new neurons. This may be less problematic in the eye, where photoreceptors derived from embryonic stem cells have been demonstrated to survive and restore light responses in congenitally blind mice [39].

The neurogenic capacity of the CNS is negatively altered after trauma. After an injury or ischemic insult, NSPCs proliferate uncontrollably and alter migratory patterns, localizing to the site of injury [40]. Several signaling cascades which boost proliferative activity have been observed in NSPCs after middle cerebral artery occlusion, including HIF1, Notch4, and EphrinB2 [41]. Elements within the microenvironment, such as blood vessels and activated microglia, play a role in establishing migration routes, by acting as scaffolding [42, 43] and releasing chemoattractants [40, 44]. In addition, altered migration may be easier for NSPCs in the injured brain due to expression of matrix metalloproteinases that digest extracellular proteins [45]. Although observations of post-injury proliferation demonstrate the plasticity of endogenous NSPCs, many cells produced do not survive. The cells that do survive assist in forming scar tissue, through which neuronal circuitry cannot readily pass. In short, although restrictions on neurogenic activity appear to be relaxed in the injured CNS, the net effect of increased proliferation, decreased cell survival, and alterations in migration is ultimately not favorable to neuronal survival or regeneration. It is not clear that transplantation of exogenous stem cells would benefit tissue repair in such an inhospitable environment, particularly that observed after ischemic stroke or spinal cord injury. Strategies have been employed to boost axonal regeneration in the adult CNS, particularly in the spinal cord, by altering adverse micro-environmental components, notably Neurite Outgrowth Inhibitor (Nogo-A), with pharmacological or antibody-based methods [46–48]. A Phase II clinical trial evaluating the safety and efficacy of anti-Nogo-A in treating amyotrophic lateral sclerosis is currently ongoing, although a similar trial evaluating this treatment in multiple sclerosis has been terminated [7].

### Modulation of endogenous neurogenesis

A multitude of behavioral, dietary, and pharmacological factors, as well as intracellular pathways, have demonstrable effects on the neurogenic activity of endogenous neural stem and progenitor cells. Studies in rodents demonstrate the incredible capacity of both environmental and intrinsic factors to regulate the proliferation, differentiation, and survival of cells in the adult mammalian brain. These factors alone are not likely to have therapeutic benefit for humans afflicted by CNS injury or degenerative disease, but such interventions may be useful to consider in combination with stem cell transplants.

Environmental enrichment increases neurogenesis, primarily in the adult hippocampal dentate gyrus [49, 50]. The effect of environmental enrichment on hippocampal neurogenesis is thought to be on promoting cellular survival; increased neurogenesis due to novelty appears to have a different mechanism than that due to cardiovascular exercise because the net neurogenic effects of these two activities together are larger than either alone [51].

The diet also influences rates of neural regeneration. In studies of rats and mice, diets high in monounsaturated fats, such as coconut oils, decrease neurogenesis [52], while those high in polyunsaturated fatty acids, such as olives, fruits, and nuts, increase adult neurogenesis [53]. Alcohol has been shown to inhibit proliferation and cause cell death in the postnatal hippocampus [54]. Yet the effect of alcohol, by gavage or voluntary intake, on neurogenic activity depends on how high the blood alcohol level is; no study achieving blood alcohol levels under 0.18% has demonstrated an inhibition of cell proliferation [55].

Chronic stress decreases neurogenic activity in the adult brain, especially in females [56], while antidepressants increase neurogenesis [57]. Stress can lead to release of corticosterone into the bloodstream by the adrenal gland. Activation of glucocorticoid receptors in hippocampal NSPCs has been shown to mediate the negative effects of corticosterone on neurogenesis [58]; adrenalectomy reverses this effect [57]. The impact of antidepressants on hippocampal proliferation, shown by increased labeling of KI67 (a cell cycle marker), has been demonstrated to be correlated with decreased plasma corticosterone [57]. Corticosterone levels therefore are considered a critical negative regulator of neurogenesis in the adult brain.

Cannabinoids have been shown to promote several aspects of neurogenesis within the adult mouse hippocampus, promoting proliferation and survival, but not differentiation, of NSPCs [59, 60]. This class of molecules include endogenously produced anandamide (AEA) and 2-arachidonylglycerol (2-AG); plant-derived cannabidiol (CBD) and delta-9-tetrahydrocannabinol (THC); and their synthetic counterparts, all of which activate either or both of the CB1 and CB2 receptors. The CB2 receptor has less widespread expression than CB1, and is found specifically in abundance on adult NSPCs in the SVZ and DG [60]. The CB2-specific agonist JWH-133 and the CB1/CB2 agonist WIN55,212-2 promote cellular proliferation *in vivo*; these drugs increase KI67+ index by 23% and 31% in the young brain, respectively, and by 350% and 600%, respectively, in the aged brain, while the CB1 agonist ACEA has no effect on neurogenesis [61]. Inhibition of fatty acid amide hydrolase (FAAH), which degrades endocannabinoids, can also promote cell proliferation *in vitro* and *in vivo*[62]. Many of these studies utilize synthetic ligands to promote neurogenesis; one recent study of naturally occurring compounds showed that CBD increased proliferation in the adult hippocampus, although another plant-derived cannabinoid, THC, decreased water maze performance and increased rotarod performance without effects on adult hippocampal neurogenesis [63].

Studies of cortical development have proved highly relevant to understanding the regulation of adult neurogenesis. Genetic knockout of *Wnt3a* during embryonic development leads to complete DG ablation [64], and mice missing other Wnt pathway elements, such as *Lrp6* or *Lef1*, or the Wnt transcriptional target, homeobox protein *Emx2*, have reductions in DG progenitors and granule neurons [65–68]. Wnt3a appears to be crucial for adult hippocampal neurogenesis as well, playing a role in both proliferation and neuronal fate commitment [69]. Other signaling pathways, such as Notch, have primarily been investigated for roles in developmental neurogenesis, although this factor too appears to play a critical role in adult neurogenesis [70, 71]. Changes in both cell-intrinsic and micro-environmental factors also affect neurogenic capacity in the aging brain, including chromosomal loss of heterozygosity [31], increased telomerase activity [72], decreased notch signaling [70], decreased wnt signaling [69], and decreased growth factors in the microenvironment [73] – the latter possibly due to decreased vascularity in the neurogenic niche [74]. Cell transplantation therapies may require genetic or epigenetic manipulation of such factors to predispose limited proliferation, successful differentiation, and effective integration into the adult CNS.

Much discussion regarding regulation of adult neurogenesis centers on the broad topic of cell-intrinsic versus cell-extrinsic influences. However distinguishing between intracellular and micro-environmental influences on neurogenesis is somewhat artificial. Cell cycle progression cannot occur without the go-ahead from extrinsic sources, such as growth factor signaling and availability of bio-energetic substrates, and cell-extrinsic signals must be read by a competent cell with downstream effectors capable of translating external signals. This view, of a singular network integrating the actions of the cell with those of the population, involves the breakdown of the concept of “self” versus “other”. Perhaps, on a biological level, this breakdown occurred at the advent of the multicellular organism. Cancer, then, can be understood in terms of a re-assertion of self (the mutated cell) at the expense of others (for example, the surrounding parenchyma and vasculature). Attaining a harmony between individual cells and the other tissues is required for the success of a multicellular organism; maintaining this harmony is key to regenerating tissue without significantly increasing the risk of cancer. To achieve the aims of regenerative medicine, new cells, whether transplanted or endogenously produced in the brain, must receive information regarding not only what they are, but what their role is within the community.

Some of the greatest progress in stem cell biology over recent decades has been in simply creating a better understanding of the molecular mechanisms involved in cellular proliferation and differentiation, through studies of rodents and cultured cells. Promoting endogenous neurogenesis will achieve only limited, localized neuron production, and is therefore insufficient for therapeutic intervention in humans, but the knowledge we have gained from studying endogenous NSPCs will undoubtedly aid the field of regenerative medicine to better guide the proliferation, fate restriction, differentiation, survival, and functional integration of transplanted cells.

### Problems [and emerging solutions] for stem cell transplants within the central nervous system

Practical translational issues encountered by prospective stem cell therapies targeted to the central nervous system include designing a clinical-grade manufacturing process, testing the therapy in an appropriate preclinical model, selecting a relevant patient population, and monitoring the transplanted cells [8]. Yet before this translational process even begins, the reluctance of the adult central nervous system to support regeneration must be considered in order to better devise stem cell-based therapeutic strategies. Below is a list detailing the challenges encountered by stem cell therapies in the central nervous system and how these concerns are being addressed.

1. **Like any transplant, rejection is a risk**

Immune activity in the central nervous system is lower than other tissues, but rejection of a transplant is still possible. Risk can be mitigated by collecting cells from the patients themselves and “dedifferentiating” the cells into a pluripotent state. Such induced pluripotent stem cells (iPSCs) individualise therapy to reduce the incidence of immune activity associated with rejection. However iPSCs require viral-mediated transduction of host cells to activate pluripotency genes, which have oncogenic properties. Non-integrating viruses or use of chemical transfection techniques avoid problems associated with genomic insertions, but oncogene reactivation and aberrant chromatin modification remain a danger regardless of the method of reprogramming. This is particularly an issue in the low oxygen environment of the CNS, as hypoxic conditions have been shown to re-induce pluripotency in fate-committed cells [75].

Bone-marrow-derived mesenchymal stem cells (MSCs) are easy to harvest from adult individuals and were briefly thought to be promising autologous sources of material to produce iPSCs, but they do not differentiate well into neuronal cell types [8]. However MSCs have been shown to secrete supportive neurotrophic factors, so co-transplantation of this cell type may still have clinical benefit. Human embryonic stem cells (ESCs) or umbilical cord blood-derived stem cells are now considered the best source of normal pluripotent cells. They can be expanded in culture, directed to differentiate into any neuronal lineage, and maintain low chromatin abnormalities.

2. **Excess proliferation can lead to cancer**

Increased NSPC proliferation can be easily and reliably achieved by knocking down tumor suppressor pathways or activating oncogenes. But this strategy transforms normal cells into cancerous ones [76, 77], so these options are not suitable means of restoring cellular content in any tissue type. Proliferative activity, particularly after transplantation, must occur in only a limited fashion. Some researchers have recently bypassed the potential problem of tumor formation arising from stem cell transplants by pre-differentiating pluripotent (or induced pluripotent) cells to an immature yet fate-restricted state prior to transplantation [6, 78, 79]. Basic research on the process of endogenous neuronal fate commitment has benefited the optimization of these protocols [24]. But pre-differentiation may not completely preclude oncogenic risk; complex chromatin modifications occur during embryonic development and dedifferentiation, so even ESCs and iPSCs that have been fate-restricted may bear hallmarks of pluripotency within their genomes. Studies are ongoing to compare the chromatin state of iPSCs, ESCs, NSPCs, and cancer cells to better understand and manage the risk of oncogenic transformation.

3. **Making a neuron to order is a difficult task**

The developing brain is capable of producing an astounding variety of neuronal subtypes. Yet the adult brain is more limited, due to a number of molecular and biophysical characteristics. It is an extraordinarily difficult task to recapitulate even a small aspect of the developmental program in the adult or aged brain. Much progress has been made in cell cultures: a number of protocols have been designed to optimize large-scale production of particular neuronal subtypes [78, 79]. The desired cell type or mix of cell types can be further enriched by sorting for surface antigens (antibody-based selection) or with fluorescent reporters (promoter/enhancer-based selection). But even if cells take on the appropriate anatomical characteristics and immunohistochemical markers, they will not necessarily function as expected if transplanted into the adult mammalian brain, eye, or spinal cord. When cells are reprogrammed, we must ask whether these cells are ‘true’ subtype-specific neurons – with the expected electrophysiological and epigenetic properties, the proper neurotransmitter phenotype and the required complement of ion channels. This issue is difficult enough when one cell type is demanded, such as dopaminergic neurons to replace those lost in Parkinson’s Disease, but the problem becomes even more challenging when more than one cell type may be needed to regenerate the tissue and reduce or bypass normal scar formation, as in the case of spinal cord injury.

4. **Controlling the influence of the micro-environment is near impossible**

The CNS generates neurons during a developmental period and does not prefer to do so later on. The adult brain microenvironment in fact encourages glial differentiation. Even if cells are pre-differentiated toward a neuronal fate, the transplant itself could cause sufficient injury to initiate glial scarring and inhibit the survival and integration of newly transplanted neurons. There is great interest in limiting or guiding the effects of the CNS environment on the transplanted cell [46–48]. However, since our understanding of the complex milieu of the adult CNS is limited, the best strategy may be to imitate structural elements of it. Establishing a conducive local microenvironment by combining transplanted cells with a simulated extracellular matrix may promote functional reorganization of the tissue [80], while introducing microgravity prior to transplantation appears to aid survival and integration of the cells [81]. In addition, neurotrophic factors injected along with the cells or secreted by the transplanted cells may work to provide a favorable environment for tissue regeneration [82]. These therapies are currently being evaluated separately and in combination with stem cell-based therapies.

5. **The fate of most transplanted cells is death**

Pioneering clinical transplantation studies endowed human Parkinson’s Disease patients with tissue grafts from up to seven fetal brains, a source and quantity that is both controversial and practically unrealistic. Cell survival was estimated to be only 5% in histopathological assessment eighteen months post-transplantation [5]. More recently, monkeys with MPTP lesions were injected with 300,000-600,000 ESCs pre-differentiated to a dopaminergic neuron state, but less than 1% of these transplanted cells remained after three months [83]. In both cases, lasting clinical benefit was reported despite the low cell survival rate, suggesting that few cells may actually need to survive in order to provide functional recovery.

6. **Forging connections in a new place can be hard**

Full neuronal differentiation *in vitro* is likely to preclude integration into the brain circuitry, because forging connections with other neurons is a critical part of neuronal maturation and cell survival. Much research has gone toward exploring intermediate states of differentiation, to manufacture implantable cells that are fate-restricted yet still ready to forge an identity within the neuronal circuitry. Self-assembling polymers provide a three-dimensional framework to aid network integration; introducing elements of the extracellular matrix may also help to cultivate nearby vasculature for metabolic support [80]. Circuit integration can be monitored by combining transplants with optogenetic stimulation, a process which may also prove useful in enhancing activity-dependent survival of newly transplanted cells [84]. In some cases however, even successful cell survival and integration of implanted cells does not guarantee functional recovery after injury [85]. Conversely, therapeutic benefit has been observed after cellular transplantation in the adult CNS even without any evidence of axonal outgrowth or improved functional connectivity, due in part to neurotrophin secretion by the transplanted cells [82]. These studies support the idea that successful survival and integration of transplanted cells into existing neural networks may not always be necessary to achieve some clinical benefit from stem cell therapies. Indeed, if some axons are spared after spinal cord injury, pro-survival and remyelination cues may be sufficient to improve recovery without the need for cell replacement [86].

7. **Immature neuronal excitability could lead to network dysfunction**

The addition of new cells, particularly immature excitatory neurons, which fire action potentials in response to the (normally inhibitory) neurotransmitter GABA, might disrupt existing neural network activity or confer novel characteristics on CNS circuitry [24]. This area of electrophysiological research is underexplored, and the risks associated with this issue are understudied in relevant preclinical models. However, the transplantation of inhibitory neuron precursors has notably been shown to decrease epileptogenesis [87].

### Regenerative medicine: a promising route?

Although some studies have shown encouraging results, a number of challenges remain to making cellular transplantation a realistic clinical treatment for neurological disorders. If stem cell therapies alone do not prove to have strong, lasting clinical benefit, combination therapies may hold promise. Cell transplantations may be supplemented by optogenetic or electrical stimulation to promote axonal remodeling. Cells might be transplanted alongside growth factors, anti-anti-growth factors, or a simulated extracellular matrix. Moreover, autologous cells may be engineered prior to transplantation to produce neurotrophic factors or even to correct a genetic deficiency, in order to support the function of an existing neural population rather than replacing it entirely.

An alternative strategy to regenerative medicine is to bypass damaged tissues completely using a neural prosthetic device or direct electrical stimulation. For example, signals from the motor cortex can be translated directly to the muscles or to a robotic arm after spinal cord injury or brainstem stroke using a brain-machine interface to achieve functional motor control [88]. And published reports demonstrate that deep-brain stimulation reliably and effectively controls symptoms of dystonia in patients with Parkinson’s Disease [89]. Stem cell-based therapies in the CNS have achieved nothing near these successes. It is important to note that the therapeutic potential of brain-machine interfaces and deep-brain stimulation was only realized after decades of basic research into the anatomy and physiology of underlying neural circuitry [90]; stem cell-based methods for treating neurological disorders must similarly comprehend the inherent properties of the CNS before attempting to fix it.

## Conclusions

Despite all of the challenges faced by stem cell therapies within the central nervous system, a number of leaps forward have been made that bring the promise of such treatments ever closer. Yet the eagerness for new therapies must always be tempered by careful assessment of the preclinical data and an understanding of the benefits and limitations of regeneration in the adult CNS. To achieve clinical therapies, we will need to address the obstacles posed by the adult CNS: namely risks associated with rejection, overproliferation, glial scarring, neuronal subtype specification, micro-environmental limitations, and difficulties in cell survival and circuit integration.

## Authors’ information

The author received her Ph.D. in Neurobiology & Behavior from the University of Washington in Seattle, USA, and currently studies neural stem cell dynamics during aging and tumorigenesis at Newcastle University in the U.K.

## Abbreviations

CNS: Central nervous system
DG: Hippocampal dentate gyrus
SVZ: Subventricular zone
NSPCs: Neural stem and progenitor cells
ESCs: Embryonic stem cells
iPSCs: Induced pluripotent stem cells
MSCs: Bone marrow-derived mesenchymal stem cells.
